# A case report of isolated primary herpes-simplex virus neuroretinitis in an immunocompetent adult

**DOI:** 10.1186/s12886-022-02272-7

**Published:** 2022-02-01

**Authors:** Víctor Lázaro-Rodríguez, Halima Berrada, María José Capella

**Affiliations:** 1 Centro de Oftalmología Barraquer, Muntaner 314, 08021 Barcelona, Spain; 2 Institut Universitari Barraquer, Universitat Autònoma de Barcelona, Barcelona, Spain

**Keywords:** Herpes simplex virus, Neuroretinitis, Primoinfection, Macular star, Case report

## Abstract

**Background:**

Herpes simplex virus (specifically HSV-1 and HSV-2) are greatly prevalent viruses that can cause conjunctivitis, keratitis and other rarer ocular disorders such as acute retinal necrosis syndrome or neuroretinitis. We report a case of an isolated unilateral neuroretinitis with primary HSV infection in an immunocompetent adult without other related clinical features.

**Case presentation:**

A 60-year-old immunocompetent woman presented with sudden painless central vision loss in her left eye (best corrected visual acuity was 20/200) showing optic disc edema, submacular fluid and a delayed development of a macular star. The macular optical coherence tomography (OCT) showed a serous retinal detachment. Arterial hypertension or exposure to ionizing radiation were ruled out and the microbiological blood test battery was only positive for immunoglobulin M (IgM) for HSV-1 which allowed etiological treatment with oral valacyclovir. Complete resolution and good visual results were found within 3 months.

**Conclusions:**

The present case of isolated neuroretinitis as a primary HSV infection in an immunocompetent patient was resolved with good functional results after valacyclovir treatment. Presence of HSV IgM in absence of other laboratory results could be enough evidence to start HSV treatment in immunocompetent patients with a macular star, as an isolated lesion, after ruling out other non-infectious causes, such as arterial hypertension or exposure to ionizing radiation. Rare infectious agents in immunocompetent patients must be considered in the differential diagnosis of neuroretinitis, even if there are no other typical symptoms or signs that could suggest the disease.

## Background

Herpes simplex viruses (specifically HSV-1 and HSV-2) are greatly prevalent. In 2016, a 66.6% of the world’s population aged 15–49 years were living with HSV type 1 infection and 13.2% with HSV type 2. The spectrum of disease includes primary and recurrent infections that causes cutaneous or genital herpes, encephalitis, conjunctivitis, and keratitis [1], establishing a lifelong latent infection with reactivations. Other ocular disorders such as acute retinal necrosis syndrome or neuroretinitis [2, 3] are rare occurrences for these pathogens. Asymptomatic primary infection is the most frequent form observed in patients with herpes simplex virus (HSV) [1].

We report the case of an isolated unilateral neuroretinitis as manifestation of a primary HSV infection in an immunocompetent adult without encephalitis or other related clinical features.

## Case presentation

A 60-year-old immunocompetent woman with no significant medical nor surgical history presented with sudden scotoma and painless central vision loss in her left eye. Her medical history revealed cataract surgery in both eyes 1 year prior and myocardial infarction 9 months prior to her visit. Best corrected visual acuity (BCVA) was 20/20 in the right eye and 20/200 in the left eye. On general examination, there were no characteristic herpetic skin lesions. On ocular examination, optic disc edema and juxtapapillary hemorrhages in the left eye were detected. There were no other signs of intraocular inflammation and the right eye features were unremarkable.

In view of its characteristics, the first approach was within an ischemic context. Arteritic and non-arteritic anterior ischemic optic neuropathy (AION) needed to be ruled out by analyzing the patient’s history, complementary tests, and medical assessments. Arteritic AION was excluded based on normal erythrocyte sedimentation rate and absence of extraocular clinical signs. The possibility of a non-arteritic AION was considered, and close monitoring was maintained.

**Fig. 1:**
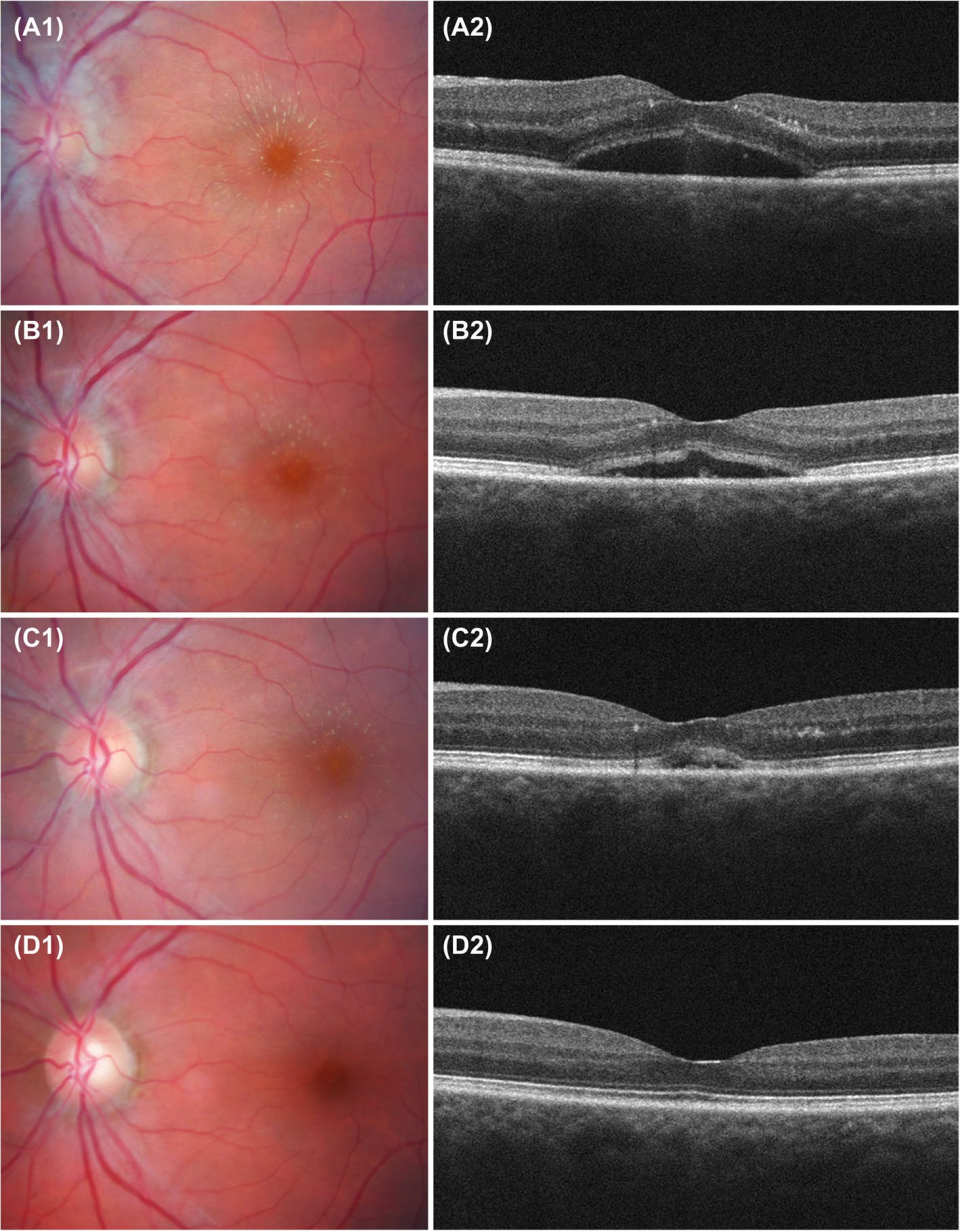
Color retinography images showing a macular star (**A1**) and its progressive reduction (**B1** at 2 weeks, **C1** at 1 month, and **D1** at 3 month). Optical coherence tomography images showing serous retinal detachment (**A2**), and its progressive resolution (**B2** at 2 weeks, **C2** at 1 month, and **D2** at 3 month)

Two weeks later, the Goldmann visual field exam revealed no abnormality. The left eye fundus revealed an optic disc edema, submacular fluid and a delayed development of a macular star (Fig. 1. A1). The macular optical coherence tomography (OCT) showed a serous retinal detachment (Fig.1 A2).

The macular star observed in the fundus helped to establish a new differential diagnosis that included diseases like hypertensive or radiation-induced retinopathy and secondary or idiopathic neuroretinitis. There was no history of arterial hypertension nor exposure to ionizing radiation. The neuroretinitis path was comprehensively analyzed and clinical and biological examinations were made.

Contact risks, including food habits, recent travel, sexual experience, and animal contacts were thoroughly explored. A detailed physical examination was performed to note sites with rashes or inoculation spots but was unremarkable. Concerning laboratory tests, immune profile, tuberculin skin test and serological evaluation were performed, including Bartonella, Epstein-Barr virus, Rickettsia, Lyme disease, histoplasmosis, syphilis, chlamydia, human immunodeficiency virus, toxoplasmosis, brucellosis, viral hepatitis B and C, HSV-1, and HSV-2. The only clinical abnormality was a positive immunoglobulin M (IgM) for HSV-1, which in absence of any other systemic clinical feature allowed etiological diagnosis.

A treatment regimen with valacyclovir 1 g every 8 h was started and we observed a clinical improvement at 2 weeks with a decrease of the symptoms and progressive resolution of the fundus signs (Fig. 1 B1, B2, C1 and C2). After 48 h of antiviral treatment, oral tapered prednisone regimen was added for 6 weeks and valacyclovir was decreased to 1 g per day until discontinuation of oral prednisone (length of antiviral treatment of 10 weeks). The treatment was well tolerated and no adverse events occurred. The patient recovered over 3 months without any sign of intraocular inflammation (Fig.1 D1 and D2) and an improvement of BCVA up to 20/25.

## Discussion and conclusions

Neuroretinitis cases have been related to a variety of infectious agents [4]. Other rare pathogens should be considered when more common etiologies such as *Bartonella henselae* have been excluded. Infectious neuroretinitis in immunosuppressed patients are usually common. However, in immunocompetent patients a few cases with mild systemic signs or symptoms that were resolved with antiviral agents have been reported. Specifically, a previously published report of a 28-year-old male with signs of neuroretinitis, chickenpox and positive titer of immunoglobulin G (IgG) for varicella zoster virus without polymerase chain reaction (PCR) analysis was completely resolved with oral acyclovir [2]. Moreover, a 48-year-old female with neuroretinitis was positive using PCR and IgG positive for both cytomegalovirus and HSV-1 with previous history of flu symptoms was successfully treated with intravenous and intravitreal ganciclovir [3]. Here, we present an unusual case of neuroretinitis in an immunocompetent patient with no other systemic symptoms or signs in a primary HSV infection.

When a patient shows typical manifestations of HSV infection, a clinical diagnosis might be enough. In an immunocompetent patient with atypical manifestations like neuroretinitis, biological examinations could be useful, such as blood tests for specific antibodies or nucleic acid amplifications tests. A PCR test is often a very useful tool to confirm a diagnosis of HSV. It is worth mentioning that, the reliability of a PCR test on ocular fluids has been reported to be lower in immunocompetent than in immunosuppressed patients [5]. Specifically, a negative PCR result cannot reliably rule out the infection in immunocompetent patients but a positive result, certainly, can confirm it. Unfortunately, a PCR test at the acute stage for our patient was not performed, so we based the diagnosis on positive IgM with negative IgG titers for HSV-1. This was also supported by the good response to antiviral treatment, as in the previously reported cases in immunocompetent patients [2, 3].

In conclusion, we report a case of isolated neuroretinitis as a primary HSV infection in an immunocompetent patient with resolution and good functional results after valacyclovir treatment. Presence of HSV IgM in absence of other laboratory results could be enough evidence to start HSV treatment in immunocompetent patients with a macular star, as an isolated lesion, after ruling out other non-infectious causes, such as arterial hypertension or exposure to ionizing radiation. Rare infectious agents in immunocompetent patients must be considered in the differential diagnosis of neuroretinitis, even if there are no other typical symptoms or signs that could suggest the disease.

## Data Availability

Not applicable.

## Abbreviations

AION: Anterior ischemic optic neuropathy
BCVA: Best corrected visual acuity
HSV: Herpes simplex virus
HSV-1: herpes simplex virus type 1
HSV-2: herpes simplex virus type 2
IgG: Immunoglobulin G
IgM: Immunoglobulin M
OCT: Optical coherence tomography
PCR: Polymerase chain reaction
